# Radiation-induced malignancies after stereotactic radiosurgery for brain arteriovenous malformations: a large single-center retrospective study and systematic review

**DOI:** 10.1007/s10143-024-03093-6

**Published:** 2024-11-26

**Authors:** Takeru Hirata, Motoyuki Umekawa, Yuki Shinya, Hirotaka Hasegawa, Atsuto Katano, Aya Shinozaki-Ushiku, Nobuhito Saito

**Affiliations:** 1 Department of Neurosurgery, The University of Tokyo Hospital, 7-3-1 Hongo, Bunkyo-ku, Tokyo, 113-8655 Japan; 2 Department of Radiology, The University of Tokyo Hospital, 7-3-1 Hongo, Bunkyo-ku, Tokyo, 113-8655 Japan; 3 Department of Pathology, The University of Tokyo Hospital, 7-3-1 Hongo, Bunkyo-ku, Tokyo, 113-8655 Japan; 4 Department of Neurologic Surgery, Mayo Clinic, 200 First St. SW, Rochester, MN 55905 USA

**Keywords:** Arteriovenous malformation, Glioblastoma, Gliosarcoma, Late radiation-induced complications, Radiation-induced malignancies, Stereotactic radiosurgery

## Abstract

Stereotactic radiosurgery (SRS) is widely utilized to treat small- and medium-sized brain arteriovenous malformations (BAVMs); however, radiation-induced malignancies (RIMs) have been reported as extremely rare yet potentially life-threatening complications of SRS. This study aimed to investigate the risk of RIMs after SRS for BAVMs. The outcomes of patients who underwent single-session SRS for BAVMs at our institution and were followed for ≥ 5 years were analyzed to calculate the incidence of RIMs. In addition, a systematic review was conducted using the existing literature reporting RIMs after SRS for BAVMs in compliance with the PRISMA guideline. Regarding the in-hospital analysis, only one (0.18%) RIM (gliosarcoma) was observed among 569 patients, with a median follow-up period of 151 months (interquartile range, 103–255 months). The 15, 20, and 25-year cumulative incidences of RIMs were 0%, 0%, and 1.01%, respectively, whereas the overall incidence rate was 0.12 per 1,000 patient-years. In the systematic review, 14 studies were included, with the incidence of RIMs ranging from 0.00 to 0.24%. Eight patients with RIMs were identified, and the most common pathology was glioblastoma. The median time until the diagnosis of RIM was 7.1 years (range, 4–19 years) after SRS, and their clinical courses were largely dismal, with the post-diagnosis survival periods being 1–10 months. RIM constitutes an extremely rare but potentially fatal complication following SRS for BAVMs, with its incidence rate being at most 0.24%.

**Supplementary Information:**

The online version contains supplementary material available at 10.1007/s10143-024-03093-6.

## Introduction

Brain arteriovenous malformations (BAVMs) are presumed to be congenital cerebrovascular disorders characterized by a nidus, which is a tangled network of abnormal arteries and veins lacking intervening capillaries. BAVMs may cause intracranial hemorrhages, headaches, epilepsy, or focal neurological deficits. Stereotactic radiosurgery (SRS), which can deliver high-dose irradiation to the nidus, is a minimally invasive procedure compared with surgical resection since it does not require general anesthesia, craniotomy, or intensive postoperative management [1]. While post-SRS hemorrhage in the latency period should be considered a rare natural negative event, SRS has been an established treatment for small- to medium-sized BAVMs, yielding an obliteration rate of 60–85% after a latency period of 3–5 years [2–4].

Numerous studies have reported the short- and medium-term efficacy and safety of SRS for BAVMs [2–6]. However, SRS has the potential risks caused by irradiation, which is sometimes irreversible. Early radiation-induced edema (known as T2-signal change) can occur in 20–30% of patients but is rarely symptomatic [3, 7]. Recent studies on the long-term outcomes (> 10 years) of SRS for BAVMs have uncovered details on late radiation-induced complications (LRICs) [1, 4, 8, 9]; cyst formation (CF) and chronic encapsulated hematoma (CEH) may, albeit rare, develop 10–15 years after SRS [8, 10, 11]. Furthermore, radiation-induced malignancies (RIMs) are extremely rare but potentially life-threatening complications [1, 4, 6, 8, 9, 12–21]. However, there remains a significant lack of data due to the scarcity of reports on RIMs. Therefore, we conducted an institutional retrospective study and systematic review to investigate RIMs following SRS for BAVMs.

## Methods

### Retrospective study

#### Patient selection

Data of 999 patients with BAVMs who underwent SRS using Gamma Knife (Elekta Instruments AB, Stockholm, Sweden) between June 1990 and January 2023 were retrospectively collected from the institutional Gamma Knife database. The exclusion criteria were as follows: (1) pre-planned staged-SRS (*n* = 33); (2) history of other radiotherapies (*n* = 29); (3) surgical resection of the nidus within 5 years from the SRS (*n* = 16); (4) follow-up period < 5 years (*n* = 352). A total of 569 patients were included in this study. The data used in this analysis were collected during hospital visits. In contrast, patients who dropped out of regular follow-up were encouraged to visit the hospital by telephone. This study was approved by the Institutional Review Board of our institution (The University of Tokyo, Clinical Research Review Board, approval number 2231, 24th January 2024), and all patients provided written informed consent. For patients treated in the past, informed consent for the study was also obtained during their visits to the hospital.

#### Radiosurgical techniques and post-SRS management

Details of the radiosurgical techniques have been described previously [22–24]. After head fixation using a Leksell frame (Elekta Instruments Inc., Stockholm, Sweden), stereotactic imaging was performed to obtain precise data on the location, shape, volume, and three-dimensional coordinates of the BAVM. Digital subtraction angiography (DSA) was performed before March 1991; thereafter, computed tomography (March 1991 to July 1996) and/or magnetic resonance imaging (MRI) (August 1996 to date) were combined with DSA to increase treatment accuracy. Board-certified neurosurgeons and radiation oncologists used commercially available software to plan treatments (KULA planning system until 1998; Leksell Gamma Plan thereafter [Elekta Instruments]). In the early cases of treatment introduction, dose selection was not clearly defined; however, at present, the marginal dose is set at 20 Gy. The nidus was precisely circumscribed using prescribed marginal doses of 20 ± 2 Gy delivered along a 50% ± 5% isodose line without any additional margins. Radiosurgical doses were meticulously adjusted based on the nidus size and its proximity to critical eloquent structures (brainstem, basal ganglia, thalamus, speech cortex with the arcuate fasciculus, and motor, sensory, and visual cortices with their pathways) [25–27]. Especially for larger AVMs exceeding 10 mL or those located in eloquent areas, dose reduction is considered. Patients were clinically and radiologically evaluated at regular intervals after SRS. MRI was performed at 6-month intervals until nidus obliteration, and DSA was performed to confirm nidus obliteration. DSA was routinely performed on all patients. If a patient declined the procedure, follow-up was conducted using only MRI. Secondary SRS was recommended for patients with a remaining nidus 5 years after the first SRS. Annual MRI examinations were performed after nidus obliteration to screen for LRICs and other complications. RIMs were defined as any tumor originating within the 20% isodose line in the irradiated area [1]. The image interpretations were conducted independently by both a neurosurgeon and a radiologist.

#### Statistical analysis

RIMs were defined using modified Cahan criteria [28–30]: (1) RIMs must arise in an irradiated field; (2) a sufficient latency period, preferably more than 3–4 years, must have elapsed between the initial radiation therapy and RIMs; (3) any primary tumor must differ histologically from the induced tumor; and (4) the tissue in which the alleged induced tumor arose must have been metabolically and genetically normal before exposure to radiation. The cumulative incidence rates of RIMs were calculated using the Kaplan–Meier method. All statistical analyses were performed using JMP^®^ Pro 17.0.0 software (SAS Institute Inc., Cary, NC, USA).

### Systematic review

#### Search strategy

This systematic review was conducted in compliance with the PRISMA guidelines. A literature search for all reports on radiation-induced neoplasms following SRS for BAVMs was conducted in June 2023 using PubMed, Cochrane, Scopus, Embase, and the Web of Science. The following MeSH terms were used: “neoplasm” OR “malignancy” OR “glioma” OR “tumor” OR “adverse events” AND “arteriovenous malformations” AND “stereotactic radiosurgery.” The reference lists of cited journals were manually searched. All identified articles were systematically assessed using the inclusion and exclusion criteria. These processes were carried out by two independent reviewers (T.H. and M.U.).

#### Eligibility criteria

The inclusion criteria comprised original research studies reporting RIMs following SRS for BAVMs. No limitations were set regarding the publication date or follow-up duration. Only articles published in English were included. Review articles, letters, editorials, comments, and technical reports were excluded, along with articles reporting vascular malformations other than BAVMs (e.g., dural arteriovenous fistulas and cavernous malformations). Regarding cohort studies from the same facility, the latest one or one with the most cases was selected.

#### Data extraction

Data on demographics, SRS treatment parameters, outcomes, and complications were extracted. The demographic data included the number of patients with AVMs treated with SRS, as well as their sex and age. Treatment parameters included nidus volume, treatment modality, and dosimetry data. Furthermore, the overall follow-up period for each study cohort and the duration until the occurrence of RIMs following SRS were also extracted. The primary outcome was the incidence of RIMs following SRS. The secondary outcomes included the number of hemorrhagic events and LRICs other than RIM.

## Results

### Retrospective study

Patient characteristics and clinical features are summarized in Table 1. The median age at the time of SRS was 34 years (range, 4–77 years), while the median follow-up duration after SRS was 151 months (range, 60–401 months). Nidus obliteration after single SRS treatment was confirmed in 426 patients (74.0%) at a median of 32 months. The 3-, 5-, 10-, and 15-year cumulative obliteration rates were 50.7%, 76.1%, 87.9%, and 91.1%, respectively, using the Kaplan–Meier method. Post-SRS hemorrhage was observed in 31 patients (5.4%) at a median follow-up of 23 months. Repeat SRS of the remnant nidus was performed in 49 patients (8.6%).

**Table 1:** Baseline characteristics and dosimetry data of 569 patients with brain arteriovenous malformations treated with stereotactic radiosurgery

Variables	Median [IQR], number (%)
Follow-up period, months	151 [103–255]
Age at SRS, years	34 [21–45]
Male sex	302 (53.1%)
Location	
Lobar location	405 (71.2%)
Basal ganglia	90 (15.8%)
Cerebellum	45 (7.9%)
Brainstem	29 (5.1%)
Eloquent location	342 (60.1%)
Deep venous drainage	295 (51.8%)
History of hemorrhage	292 (51.3%)
Planned target nidus volume, mL	2.6 [0.9–5.9]
Margin dose, Gy	20 [20–20]
Central dose, Gy	40 [40–44]
History of embolization	70 (12.3%)
History of direct surgery	52 (9.1%)
Spetzler-Martin grade	
I	116 (20.4%)
II	185 (32.5%)
III	217 (38.1%)
IV	46 (8.1%)
V	5 (0.9%)
Repeat SRS	49 (8.6%)
mPRAS	1.13 [0.79–1.54]
VRAS	
0	35 (6.1%)
1	132 (23.2%)
2	212 (37.3%)
3	138 (24.3%)
4	52 (9.1%)

IQR, interquartile range; mPRAS, modified Pittsburgh radiosurgery-based arteriovenous malformation grading scale; SRS, stereotactic radiosurgery; VRAS, Virginia radiosurgical arteriovenous malformation grading scale

In this study, only one patient (0.18%) developed RIMs following SRS. The 283-month cumulative incidence of RIM development and the cumulative incidence after 283 months were 0% and 1.01%, respectively, calculated using the Kaplan–Meier method (Supplementary Fig. 1). The total follow-up period was calculated to be 8,366 patient-years, and the overall incidence rates of RIMs after SRS for BAVMs were 0.12 per 1,000 patient-years for patients with follow-up periods of ≥ 5 years. The mean observation period of 569 patients before BAVM treatment, calculated between diagnosis and any interventions, including resection, endovascular therapies, and SRS, was 43 months. The total observation period before treatment was calculated as 2,038 patient-years, and no malignancy was detected during these periods; therefore, the occurrence of malignancy concomitant with BAVMs in natural history was 0.00% per 2,038 patient-years.

**Fig. 1:**
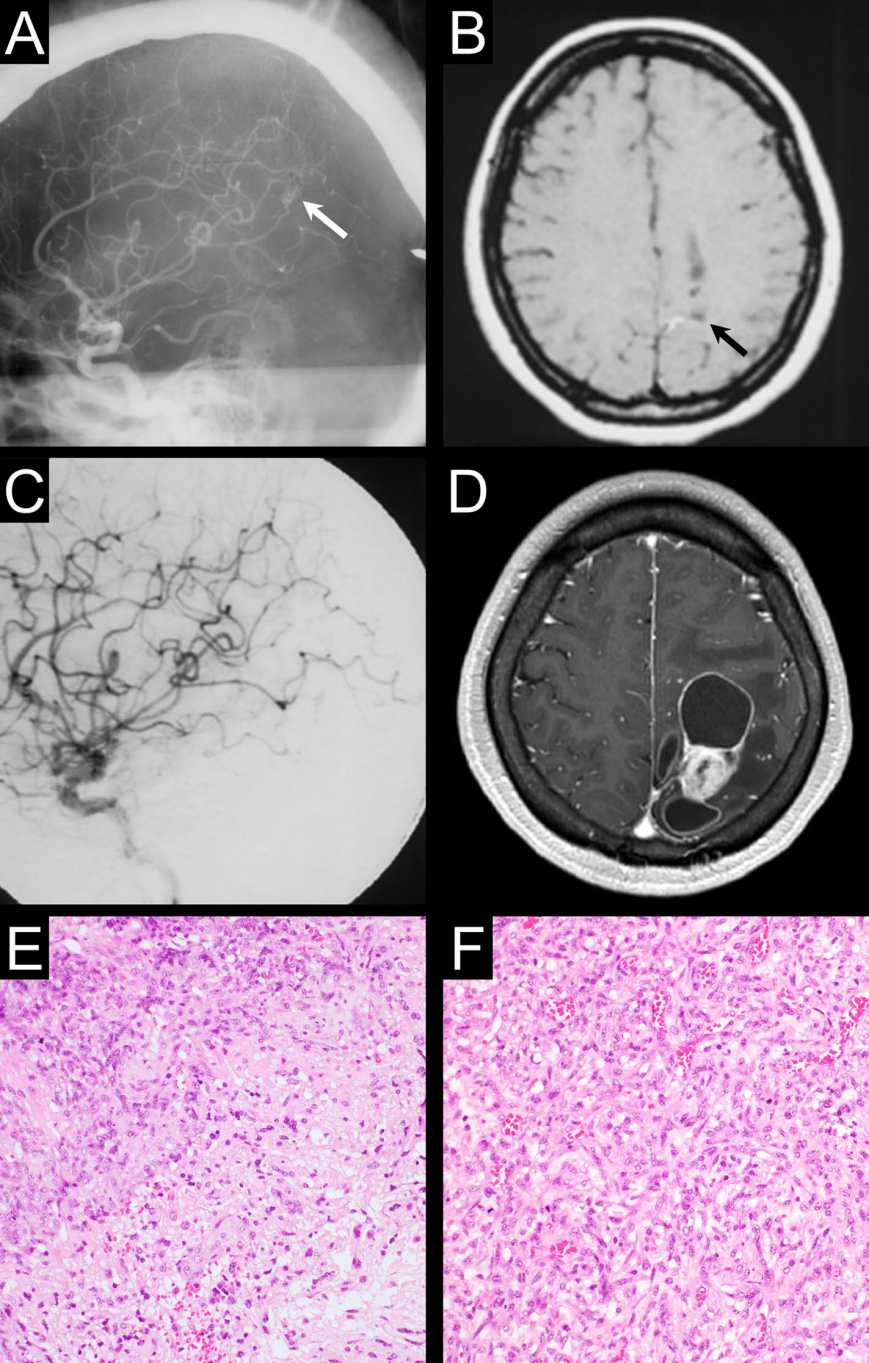
Case description of radiation-induced malignancy development following stereotactic radiosurgery for brain arteriovenous malformation. Only one patient developed radiation-induced malignancy (RIM) after stereotactic radiosurgery (SRS), accounting for a crude incidence of 0.18%. A 42-year-old female presented with intracranial hemorrhage caused by deep-seated parietal lobe brain arteriovenous malformation (BAVM) rupture. Pre-SRS angiography and enhanced magnetic resonance imaging (MRI) showed a small nidus (**A**, white arrow) and a hemorrhagic scar (**B**, black arrow). Nidus obliteration was confirmed using angiography 12 months post-SRS (**C**). After 283 months, she rapidly developed Gerstmann syndrome, and an enhanced MRI showed multi-cystic lesions near the lateral ventricle (**D**). She underwent surgical resection, and the tumor was pathologically diagnosed as a gliosarcoma characterized by gliomatous (**E**) and sarcomatous (**F**) components (Hematoxylin and eosin, original magnification x200). After resection, chemoradiotherapy was administered, and the tumor was temporarily controlled; however, the patient developed massive tumor recurrence and died three years post-SRS

The patient who developed RIMs was a 42-year-old female referred to our institution for radiosurgical treatment of a ruptured small BAVM in the left primary sensory cortex with a deep draining vein, classified as Spetzler–Martin grade III (Fig. 1A, B). SRS using Gamma Knife was performed at the nidus, with a maximum diameter of 10 mm, 0.4 mL volume, and a marginal dose of 25 Gy to the 60% isodose line. No additional surgeries or endovascular treatments were performed. Nidus obliteration was confirmed using enhanced magnetic resonance imaging (MRI) and angiography 12 months post-SRS (Fig. 1C). The patient did not develop any radiation-induced complications until 283 months. However, she presented to another hospital with Gerstmann syndrome and an expanding space-occupying lesion within the isodose line (Fig. 1D). The patient underwent surgical resection of the tumor and was diagnosed with gliosarcoma, characterized by gliomatous (Fig. 1E) and sarcomatous (Fig. 1F) components with a Ki-67 labeling index of 15.3%. She subsequently underwent chemotherapy (temozolomide and bevacizumab) and extended-local radiotherapy with 60 Gy in 30 fractions. Her symptoms were temporarily relieved; however, the tumor recurred rapidly in the parenchyma and ventricles, and she died three years after tumor detection.

### Systematic review

**Fig. 2:**
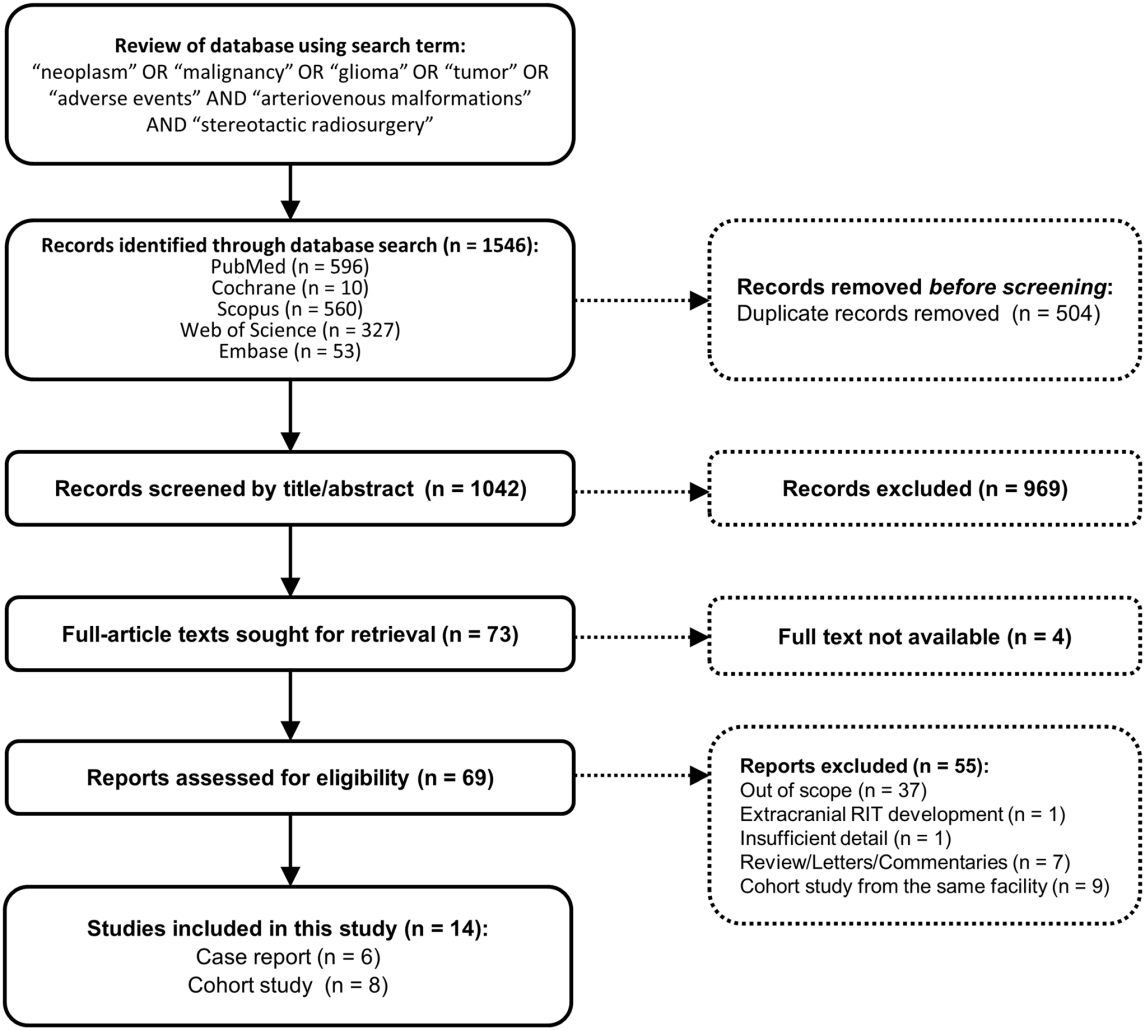
PRISMA flow diagram showing the selection process for studies included in analyses of radiation-induced malignancies (RIMs) following stereotactic radiosurgery (SRS)

An overview of this study is illustrated in Fig. 2. A literature database search yielded 1,546 studies. Most studies were excluded due to the following reasons: (1) duplication (504 articles); (2) not relevant to RIMs after reviewing the title and abstract (969 articles); (3) unavailability of the full text to review (4 articles); (4) did not meet the inclusion criteria after full text review (55 articles). Overall, 14 articles were eligible for this study, including 8 cohort studies and 6 case reports. Table 2 summarizes baseline characteristics and dosimetry and outcomes of previous cohort studies evaluating RIMs after SRS for BAVMs [6, 16, 19, 31–35]. RIMs were defined using the Cahan criteria in three studies [6, 16, 33] and any subsequent intracranial malignancy in one study [19] and were not described in four studies [31, 32, 34, 35]. The incidence of RIMs is summarized in Table 2. The incidence of RIMs was 0% in six studies [19, 31–35] and ranged from 0.08 to 0.24% in the remaining two studies [6, 16]. Tumor pathology included glioblastoma, anaplastic oligodendroglioma, angiosarcoma, high-grade glioma, and anaplastic astrocytoma and was managed using surgical resection in all patients. Details of these patients are described below.

**Table 2:** Summary of studies investigating the incidence of radiation-induced malignant tumors after stereotactic radiosurgery for brain arteriovenous malformations

Authors, year	*N* (female, %)	Median age	Median volume, mL	Previous hemorrhage	Treatment modality	Median prescription dose, Gy	Median follow-up periods, months	Complete obliteration	Post-SRS hemorrhage	Mortality	Number of RIMs	Tumor pathology
Bollet et al., 2004 [31]	118 (53%)	35^†^	6.8	62 (53%)	LINAC	17	44	60/112 (54%)	7/116 (6%)	0 (0%)	0 (0%)	
Kano et al., 2012 [32]	105 (50%)	31	6.4	43 (41%)	GK	18	80	65 (62%)	24 (23%)	9 (9%)	0 (0%)	
Kano et al., 2012 [35]	135 (44%)	12	2.5	87 (64%)	GK	20	71.3	97 (72%)	8 (6%)	4 (3%)	0 (0%)	
Starke et al., 2014 [16]	1,309 (47%)	34	2.8	NA	GK	21	94	NA	NA	NA	1 (0.08%)	Anaplastic astrocytoma
Pollock et al., 2017 [33]	233 (55%)	38	5.1	NA	GK	18	120	NA	NA	NA	0 (0%)	
Wolf et al., 2019 [19]	1,089 (48%)	35	NA	NA	GK	20	97	NA	NA	NA	0 (0%)	
Borius et al., 2022 [34]	22 (50%)	44^†^	1.3	15 (68%)	GK	18	174	20 (91%)	0 (0%)	NA	0 (0%)	
Hasegawa et al., 2022 [6]	1,249 (39%)	33	2.5	762 (61%)	GK	20	61	662 (60%)	86 (7%)	28 (2%)	3 (0.24%)	Glioblastoma, anaplastic oligodendroglioma, angiosarcoma
Present study	550 (48%)	34	2.6	287 (52%)	GK	20	148	421 (77%)	26 (5%)	13 (2%)	1 (0.18%)	Gliosarcoma

^†^Mean value

BAVM, brain arteriovenous malformation; GK, Gamma Knife; N, number; NA, not available, RIMs, radiation-induced malignancies; SRS, stereotactic radiosurgery

Table 3 shows the details of the reported RIMs after SRS for BAVMs, described in six case reports and three cohort studies [4, 6, 13, 16–18, 21, 36]. Glioblastoma was the most common type of RIM (71.4%). Pathological examination revealed infiltrating glial neoplasms comprising highly atypical cells, with some tumors demonstrating mitosis, palisading necrosis, and vascular proliferation. Immunohistochemistry results demonstrated wild-type isocitrate dehydrogenase with no isocitrate dehydrogenase mutant variants and also revealed staining for P53, which was concomitant with vimentin and glial fibrillary acidic protein, confirming glial lineage [18, 36]. Ki-67 staining showed high proliferative activity in most tumors [4, 6, 13, 16–18, 21, 36]. The Ki-67 index was mentioned in five patients; the value was noted in two (up to 50% [17] and 95–100% [18], respectively) and was described as “very high” in three. The median time for the diagnosis of RIM following SRS was 7.1 years (range, 4–19 years) across these studies. Seven RIMs were detected based on the patient’s neurological symptoms, whereas details were not described in one case. Surgical resection was performed in all patients for the initial treatment of RIMs, followed by chemotherapy in four patients and radiation therapy in the remaining four. Chemotherapy consisted of temozolomide in three patients, temozolomide plus bevacizumab in one, and nitrosourea and interferon β in one. The clinical courses were described in five patients, of whom four died, and one was transferred to hospice care. The median overall survival following RIM diagnosis was 4 months (range, 1–10 months). A detailed clinical course was described in three patients: two died in the early postoperative period (one and four months after surgery, respectively), and one was treated with postoperative chemotherapy, which was ineffective, and died 10 months postoperatively.

**Table 3:** Summary of patients who developed radiation-induced malignant tumors after stereotactic radiosurgery for brain arteriovenous malformations

Author, year	Age/sex	Previous hemorrhage	Previous treatments	BAVM location	SMG	Nidus volume, mL	Radiosurgical modality	Maximum dose, Gy	Prescription dose, Gy	Pathologies of RIMs	Latency periods after SRS, years	Treatment for RIMs	Clinical course, survival
Kaido et al. 2001 [21]	14/M	Yes	VPS	Parietal lobe	III	8.8	GK	40	20	Glioblastoma	6.8	Surgery, CT	Died (10 months after surgery)
Berman et al. 2007 [13]	34/F	Yes	None	Pineal region	NA	20.3	LINAC	21	15	Glioblastoma	9	Surgery, CRT	NA
Yoshida et al. 2014 [17]	5/F	Yes	None	Thalamus	III	NA	GK	32	16	Glioblastoma	5.8	Surgery	Died (1 months after surgery)
Starke et al. 2014 [16]	26/M	No	Embolization, VPS	Cerebellum-midbrain	IV	4.0	Proton therapy/ GK	8.5/19	12	Anaplastic astrocytoma	19	Surgery, CRT	NA
Xhumari et al. 2015 [18]	21/F	Yes	None	Frontal lobe	NA	21	CK	13	10	Glioblastoma	6	Surgery, CRT	NA
Hasegawa et al. 2019 [4]	12/F	N/A	N/A	Cerebellum	NA	2.2	GK	NA	24.8	Anaplastic oligodendroglioma	7.3	Surgery	Died (4 months after surgery)
Hasegawa et al. 2022 [6]	28/F	N/A	N/A	Occipital lobe	NA	14.1	GK, staged-GK	NA	18, 16/17	Angiosarcoma	13	NA	Died
Richter et al. 2022 [36]	63/F	No	Embolization	Frontal lobe	IV	NA	GK	46	23	Glioblastoma	4	Surgery, RT	Terminal care
Present study	42/F	Yes	None	Parietal lobe	III	0.4	GK	42	25	Gliosarcoma	24	Surgery, CRT	Died (36 months after surgery)

BAVM, brain arteriovenous malformation; CK, Cyber Knife; CRT, chemoradiotherapy; CT, chemotherapy; F, female; GK, Gamma Knife; M, male; N, number; NA, not available; RIMs, radiation-induced malignancies; RT, radiation therapy; SMG, Spetzler-Martin grade; SRS, stereotactic radiosurgery, VPS,

## Discussion

Radiation-induced tumors following radiotherapy for BAVMs are rare but potentially severe delayed complications. The risk of radiation-induced tumors (benign and malignant) after conventionally fractionated radiotherapy treatment is 1–4% after 10–20 years, which may increase based on the radiation dose [5, 37, 38]. A retrospective multicenter cohort study reported that RIMs following SRS for BAVMs, trigeminal neuralgia, or benign intracranial tumors had an overall incidence of 6.80 per 100,000 patient-years or a cumulative incidence of 0.045% over 10 years [19], which is similar to the risk of developing a malignant central nervous system tumor in the general population. Based on our cohort and the available literature, we estimated the incidence of RIMs after SRS for BAVMs at approximately 0.00–0.24% [6, 16]. This incidence is slightly higher than the reported overall incidence of RIMs following SRS for any pathology in previous studies, partially owing to the intra-axial location of BAVMs, which results in greater radiation-induced injury of the brain parenchyma [4]. Conversely, the incidence of RIMs following SRS for BAVMs may be lower, considering that several studies reported an incidence of 0% [19, 31–35, 39–42]. Previous studies have estimated the risk of radiation-induced tumors to be 0.4–1.1% in pediatric patients with BAVMs [4, 5, 14, 20, 43]. Hasegawa et al. reported the 10-year cumulative incidence of RIM development to be 1.6% in children and adolescent patients [4]. In our summary of the RIM cases, the median age at the time of SRS was 26 years old, with 33.3% aged < 20 years. Most SRSs for BAVMs are performed in adults over 20 years of age [6, 16, 19, 31–35]; hence, these results may imply that younger patients undergoing SRS experience more RIMs.

As the period between radiation exposure and tumor formation is a major criterion for radiation-induced tumors, prediction of the progression of RIMs following SRS has been debated [28]. Burke et al. reported that the incidence of radiation-induced tumor formation was not statistically different between patients with early versus late BAVM obliteration [43]. We found that the median and mean time for the development of a secondary malignant brain tumor was 7 and 11 years, respectively, with a range of 4–24 years [4, 6, 13, 16–18, 21, 36], which might be longer than that for LRICs [7, 44–49]. RIMs may develop > 20 years after SRS, as in our case, suggesting the necessity of longer follow-up periods.

In terms of morphological and dosimetrical aspects, the following characteristics of SRS for BAVMs related to RIM might be considered: (1) larger BAVMs (with a nidus volume of 9–21 mL) [6, 13, 18, 21], (2) multiple irradiations [6, 18], and (3) higher prescription dose (with 23–25 Gy) [4, 36]. In larger BAVMs, the volume of normal brain tissue surrounding the nidus exposed to irradiation increases, and multiple irradiation sessions raise the radiation burden on the surrounding normal brain. Even if the nidus is small, higher radiation doses can increase the volume of surrounding brain tissue exposed to irradiation. These facts might indicate that unnecessary irradiation of the brain should be avoided, meticulous irradiation confined to the nidus is justified, and further research into dose optimization is warranted.

In our analysis of previously reported patients with RIMs, we described the detailed clinical courses of three patients, all of whom died within one year. In contrast, our patient had no tumor recurrence for three years. The three previously reported cases included two glioblastomas and one anaplastic oligodendroglioma. The tumors were undifferentiated, had high cellularity, and tended to have a high Ki-67 labeling index. In contrast, our case consisted of gliosarcoma, characterized by gliomatous and sarcomatous components, with a Ki-67 labeling index of 15.3%. Ki-67 is considered a valuable prognostic factor for glioma; these pathological features might have affected the prognosis [50]. Additionally, there is no known effective treatment for RIMs. In general, the extent of surgical resection is a prognostic factor in glioblastoma [51]; however, although tumor resection was performed and pathological specimens were obtained in all cases of previously reported RIM patients, data regarding the degree of surgical removal of RIMs remain lacking. In addition, radiotherapy and temozolomide are the standard glioblastoma treatments [52], and RIMs are often similarly treated. Unfortunately, these treatments are not sufficiently effective for longer survival in almost all patients with RIMs.

Moreover, no radiological or histopathological features that distinguish RIMs from spontaneous malignant tumors have been identified [53–55]; however, some differences may become apparent with the evolution of genetic data analysis [56]. Radiation-induced glioblastomas had a lower rate of *EGFR* expression and incidence of p16 alterations than spontaneous glioblastomas in previous studies [57]. Furthermore, *PTEN* tumor suppressor gene mutations are frequently absent in radiation-induced gliomas [57]. In our systematic review, we found only a few genetic analyses of RIMs following SRS for BAVMs. Future studies should clarify the genetic background of RIMs following BAVM and the differences from de novo gliomas. Patients with de novo glioblastomas with a methylated *MGMT* promoter benefited from temozolomide [58], whereas those with *TERT* unmethylated mutant-*MGMT* had a poorer prognosis. Glioblastoma remains the most common pathology among RIMs, and examination of these genetic mutations may aid in prognosis prediction and evaluation of temozolomide efficacy.

Our study had a few limitations. This was a single-center retrospective study, and patient selection and radiosurgical techniques might have influenced the results. Tumor development might not have been completely detected owing to loss of follow-up. Death of patients without long-term follow-up might be a competing risk for RIMs. The low incidence of RIMs might be affected by reporting bias. Analyzing the pooled data to identify the factors linked to RIM occurrence in the systematic review was not possible due to the lack of data on individual patients, particularly those who did not develop RIMs. Some of the reported studies included insufficient follow-up, which may have led to an underestimation of the incidence of RIMs. Therefore, longer follow-ups and further investigation in multiple institutions are necessary to understand the mechanism underlying oncogenesis and the optimal treatment for this rare phenomenon.

## Conclusions

The incidence of RIMs following SRS for BAVM was extremely low (0.00–0.24%), with a latency period of 4–25 years following SRS. RIMs might be characterized by larger nidus, multiple irradiations, and higher doses of irradiation. The predominant pathology of RIMs was glioblastoma, and RIMs are likely fatal, with a survival period of 36 months or less.

## Electronic supplementary material

Below is the link to the electronic supplementary material.

Supplementary Material 1

## Data Availability

No datasets were generated or analysed during the current study.
