# Supplementary figures and images for: Radiation-induced malignancies after stereotactic radiosurgery for brain arteriovenous malformations: a large single-center retrospective study and systematic review
Source: Neurosurg Rev. 2024 Nov 26;47(1):870. doi: 10.1007/s10143-024-03093-6 (PMC11588909; doi:10.1007/s10143-024-03093-6)

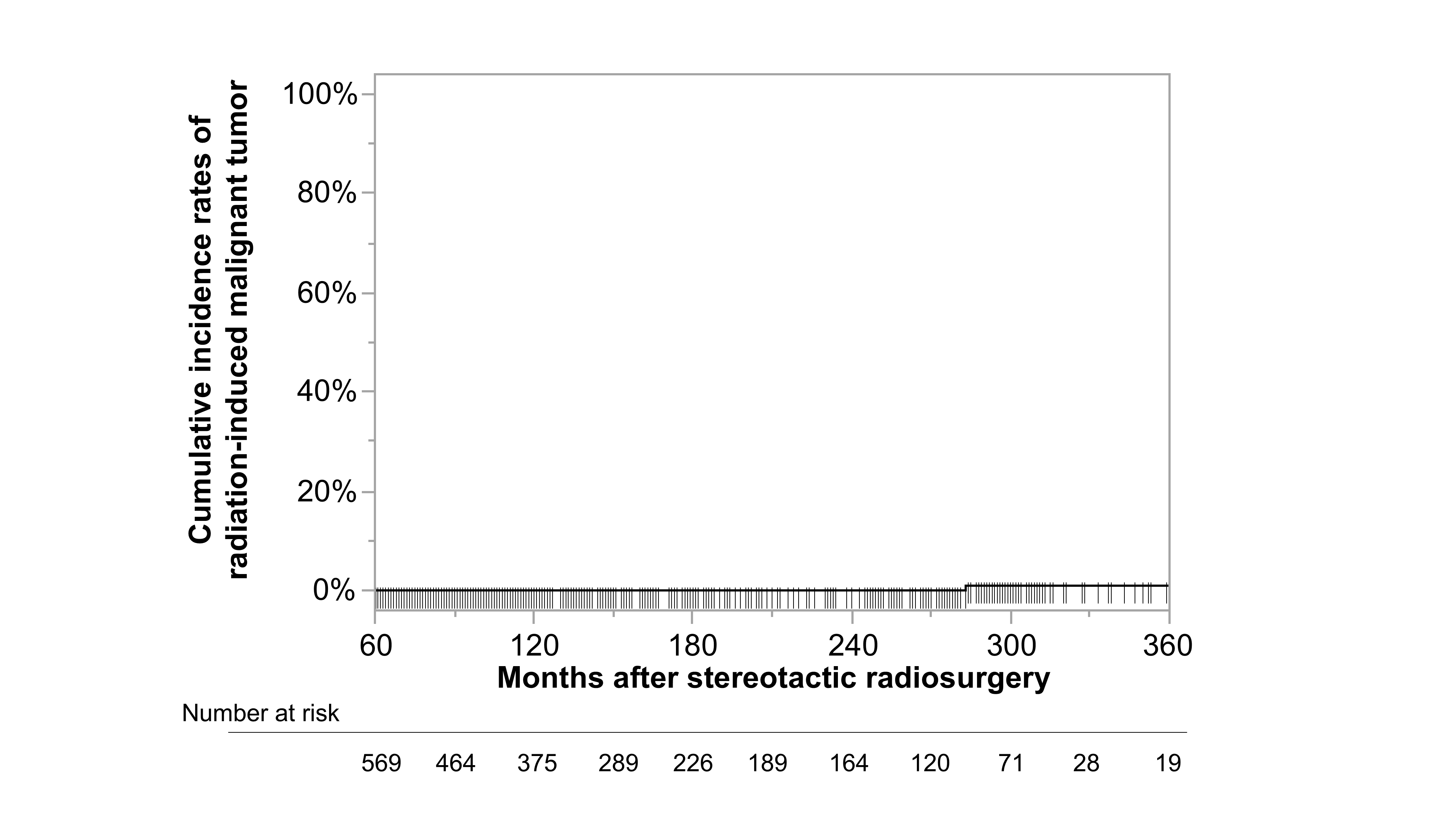

Supplement: Supplementary file 1 — Supplementary Material 1 [file 10143_2024_3093_MOESM1_ESM.tif]
